# Biogenic Silver Nanoparticles as a Stress Alleviator in Plants: A Mechanistic Overview

**DOI:** 10.3390/molecules27113378

**Published:** 2022-05-24

**Authors:** Fozia Abasi, Naveed Iqbal Raja, Zia Ur Rehman Mashwani, Muhammad Shoaib Amjad, Maria Ehsan, Nilofar Mustafa, Muhammad Haroon, Jarosław Proćków

**Affiliations:** 1 Department of Botany, PMAS Arid Agriculture University, Rawalpindi 46300, Pakistan; drnaveedraja@gmail.com (N.I.R.); zia.botany@gmail.com (Z.U.R.M.); 14-arid-4355@student.uaar.edu.pk (M.E.); nilofar_abbasi@hotmail.com (N.M.); 2 Department of Botany, Women University of Azad Jammu and Kashmir Bagh, Azad Kashmir 12500, Pakistan; malikshoaib1165@yahoo.com; 3 National Key Laboratory of Crop Genetic Improvement, Huazhong Agricultural University, Wuhan 430070, China; muhammadharoon786@webmail.hzau.edu.cn; 4 Department of Plant Biology, Institute of Environmental Biology, Wrocław University of Environmental and Life Sciences, Kożuchowska 5b, 51-631 Wrocław, Poland

**Keywords:** biosynthesis, AgNPs, environmental stresses, reactive oxygen species, ROS

## Abstract

Currently, the growth and yield of crops are restrained due to an increase in the occurrence of ecological stresses globally. Biogenic generation of nanomaterials is an important step in the development of environmentally friendly procedures in the nanotechnology field. Silver-based nanomaterials are significant because of their physical, chemical, and biological features along with their plentiful applications. In addition to useful microbes, the green synthesized Ag nanomaterials are considered to be an ecologically friendly and environmentally biocompatible method for the enhancement of crop yield by easing stresses. In the recent decade, due to regular droughts, infrequent precipitation, salinity, and increased temperature, the climate alternation has changed certain ecological systems. As a result of these environmental changes, crop yield has decreased worldwide. The role of biogenic Ag nanomaterials in enhancing methylglyoxal detoxification, antioxidant defense mechanisms, and generating tolerance to stresses-induced ROS injury has been methodically explained in plants over the past ten years. However, certain studies regarding stress tolerance and metal-based nanomaterials have been directed, but the particulars of silver nanomaterials arbitrated stresses tolerance have not been well-reviewed. Henceforth, there is a need to have a good understanding of plant responses during stressful conditions and to practice the combined literature to enhance tolerance for crops by utilization of Ag nanoparticles. This review article illustrates the mechanistic approach that biogenic Ag nanomaterials in plants adopt to alleviate stresses. Moreover, we have appraised the most significant activities by exogenous use of Ag nanomaterials for improving plant tolerance to salt, low and high temperature, and drought stresses.

## 1. Introduction

Climate change and variability are an important risk to the agricultural sector worldwide. Therefore, it is widely accepted that changes in rainfall patterns, temperature, sea water level, and CO_2_ concentration in the atmosphere will have the most devastating impacts on agricultural production [1]. It is undeniable that climate change badly affects both quantity as well as quality of crops. Anthropogenic activities mainly trigger climate change resulting in biotic and abiotic stresses such as drought, salinity, microbial diseases, and other challenges. These stresses influence plant growth and development. There is an urgent need to cope and address these challenges with infallible approaches and techniques for security, sustainability, and resilience of crops [2]. The climate pressure on agriculture results in high temperatures that lead to drought and heat waves and various biotic stresses, such as microbial and fungal stresses [3,4]. Furthermore, these irresistible plant stressors may have a global impact on crop quality and their yield. As a result, these abiotic and biotic stressors have a negative impact on agricultural yields and induce the increase of different heavy metals in plant tissues, making them unsafe for animals and people and causing significant health problems [5]. In addition, biotic and abiotic stressors have an adverse effect on the development and growth of horticulture and agricultural crops [5]. Environmental stresses are responsible for pollen sterility, altered photosynthetic and respiratory enzymes, dried seeds, and lead to an excess production of reactive oxygen species (ROS) that cause harmful impact on cell membrane, lipid metabolisms, protein content, and finally increase oxidative stress in plant species [6]. An emerging field of the 21st century is nanotechnology, which generates a highly appreciated influence on the livelihood of mankind, economy, and industry worldwide through the introduction of nanomaterials, nanorods, nano-tubes, nanosensors, nano drugs, etc., [7,8]. Nanomaterials comprise features depending on size, more surface area to volume ratio, and distinct optical properties that result in exceptional physical and biochemical specialties.

Among many applied techniques, nanotechnology is considered as one of the imperative agricultural applications and has been in practice from many years. It is deemed that nanomaterials help to improve yield by maintaining nutrient loss during fertilization through the optimization of nutrients. Nanotechnology devices and tools, such as nanocapsules, nanoparticles, and even viral capsid have been used [9]. Various discoveries and products that incorporate modified nanomaterials into farming practices, such as nanosensors, nanofertilizers, and nano-based pesticides, have been conventional with the purpose of improving the excellence of agricultural [10,11]. Silver nanoparticles are among the engineered nanomaterials that are rapidly being created and active in a wide range of consumer products. According to the US consumer product catalogue, 435 of the 1814 nano-enabled items (about 24%) include silver nanoparticle [12,13]. Nowadays, various metal-based nanomaterials, including silver nanoparticles, have gained important focus because of their eco-friendly applications in the farming sector. However, a greener method for silver NPs has been extensively utilized using extracts of plant parts in the agronomic division [14,15].

**Figure 1:**
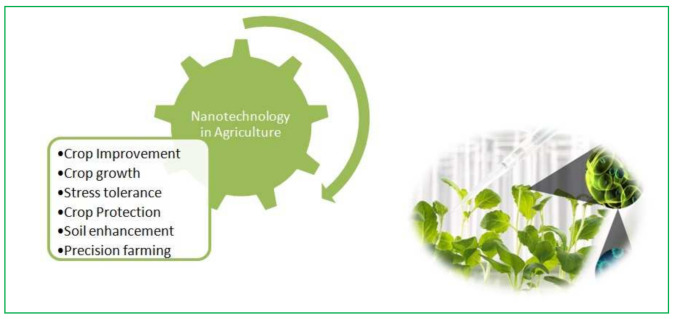
Various applications of nanotechnology in agriculture. Created with Biorender.

Worldwide, food safety is highly influenced by changing environmental conditions and increased growth rates. Abiotic stresses such as salinity, drought, low and high temperature are the most important concerns that adversely influence the growth, development, and production of plants [16,17,18,19]. More than a few metal-based nanoparticles such as Ag, Au, Fe, Cu, TiO_2_, Zn, and ZnO, have recently been used for germination, growth, and tolerance to stress of cereal crops [20]. The influence of plant-based silver nanoparticles (AgNPs) has been well-known in the agricultural sector, along with a focus on seed germination, growth, development, and gas exchange rate during abiotic and biotic stresses [21,22,23,24] (Figure 1). AgNPs have an unusually governing performance over all other NPs due to their distinctive physiological and chemical properties showing antioxidant and antimicrobial activities [25,26]. The objective of this article is to accurately analyze the underlying mechanistic resistance to stress and increased tolerance through the antioxidant activity of plant-based silver nanoparticles [27,28,29]. However, certain experiments on preparation and their characterization methods have been described in the literature; however, comparatively few studies have been performed on the greener synthesis along with tolerating effects against stresses such as abiotic and biotic in plants.

This review focuses on the green synthesis approaches with special emphasis on the plant extract-mediated synthetic approaches, and varied application potential of biogenic AgNPs in agriculture. As a result, the current review focuses on the possible applications of nanoparticles in agriculture, specifically on the influence of various NPs on crops, particularly on their growth and the reduction of abiotic and biotic stresses in plant species to accomplish the sustainable agriculture.

## 2. Green Synthesis

**Figure 2:**
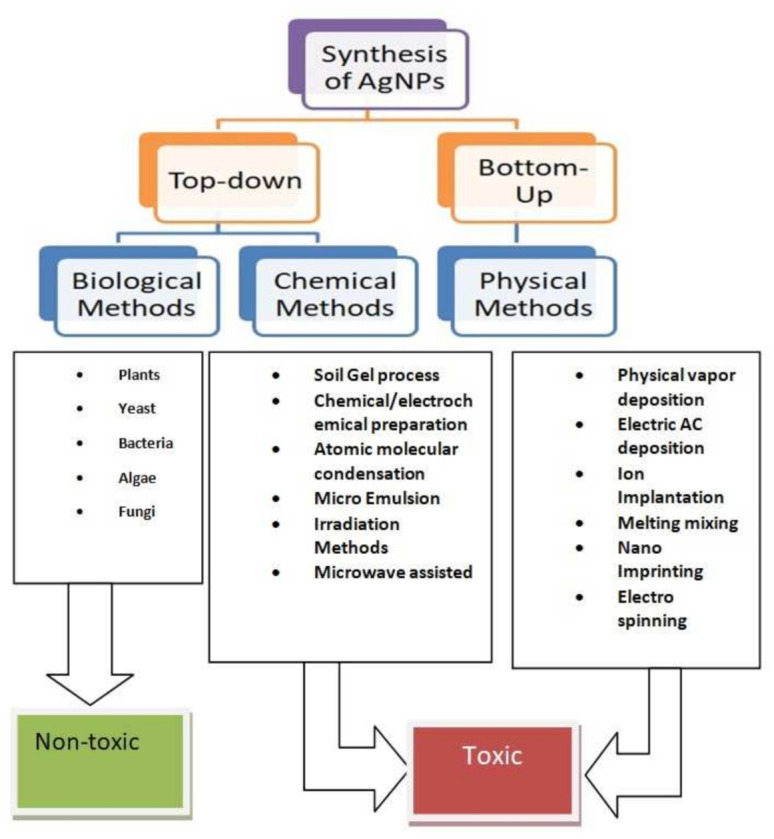
Different method used for the synthesis of (AgNPs) nanoparticles.

Traditional techniques of synthesizing nanoparticles are costly, toxic, and hostile to the environment. To address these difficulties, researchers must identify the precise green pathways, or naturally existing bases and resources that may be utilized to synthesize nanoparticles. Green synthesis may be classified into three types: (a) The usage of plants and their extracts; (b) the use of microbes such as yeasts, bacteria, and actinomycetes (prokaryotes); and (c) use of membranes and their viral DNA. This review addresses the synthesis of AgNPs using chemical, physical, and biological methods. The different methods used for the synthesis of nanoparticles are represented in Figure 2.

### AgNPs Synthesis from Plants/Microoragnisms

**Figure 3:**
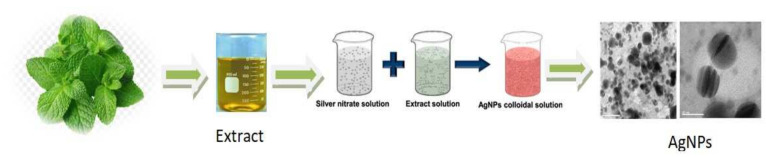
Synthesis of silver nanoparticles (AgNPs) using plant extracts.

Plants extracts have acquired appeal in green synthesis because of their rapid development, single-step technique, non-pathogenicity, profitable procedure, and ecologically benevolent nature. Plant-based green synthesis is often faster than that of other microbes, such as bacteria and fungus. As a result, the utilization of plant-based extract in green synthesis has spurred several investigations and studies. Following the selection of the plant extract, the main influencing criteria are the concentration of the extract, the metal salt, the temperature, and the pH [30]. In addition to development situations, the plant from which the extract will be taken is the most essential factor. Plants used for NP synthesis that have the advantage of being easily accessible, are treated as safe, and have an extensive range of vigorous compounds that help decrease silver ions. For the biosynthesis of NPs, the leaves, bark, shoots, stem, etc., and their derivatives are effectively used [31] (Figure 3). The limited active agents in this element, which allow for the reduction and stabilization, as well as the biomolecules that produce stable nanoparticles, are crucial. The main chemicals that impact reducing and capping NPs include biomolecules such as polysaccharides, amino acids, proteins, and alkaloids. Similarly, chlorophyll pigments, methyl chavicol, vitamins, ascorbic acid, and other elements have also been studied [32].

*Medicago sativa* shoots have been the primary choice for the production of metallic nanoparticles, as well as present the first description for the production of AgNPs from silver nanoparticles in a live plant system [33]. The usual method for producing NPs is to assemble the necessary plant part/material from accessible sources, which is followed by complete washing and rinsing with purified water [34]. Prior to mashing through a domestic blender, the plant samples were dried in dark for a period of 10–15 days. Then 10 g dry powder of the plant sample is boiled until the preparation of broth in distilled water. After that, the solution was filtered to ensure complete removal of the unsolvable particle from the broth. Then the remains were stored for a short time until its last treatment with 1 mM AgNO_3_.

The solution is then stirred in an incubator shaker where the solution changes its color as the concentration of AgO decreases, and the pure Ag+ resultant solution sample was detected at constant intervals in the ultra violet spectrum to identify the distinctive characteristics of the nanoparticles. To characterize the synthesized nanoparticles, a variety of methods must be used [34]. High resolution transmission electron microscopy, UV–Vis spectrophotometer, energy dispersive X-ray spectroscopy, and particular area diffraction were used to analyze the AgNPs synthesis using *Ananas comosus* (Bromeliaceae). Transmission electron microscopy micrographs showed spherical NPs with an average diameter of 12 nm. Chicalote leaf extract (Papaveraceae) was added to an aqueous solution of AgNO_3_, it is utilized as a capping and reducing agent in the synthesis of silver nanoparticles (AgNP). The properties nanoparticles were investigated by using a UV–Vis spectrometer, an X-ray diffract meter (XRD), a scanning electron microscope (SEM), and a Fourier transmission infrared spectrophotometer (FTIR) which revealed that the average size of NPs is 30 nm [35]. According to others, silver nanoparticles can be synthesized from a peanut shell and coupled with marketable silver nanoparticles (AgNPs) based on their antifungal activity [36]. Examination of the UV-Vis spectra, and FTIR and XRD peaks verified the similarity of the commercial and produced nanoparticles. These data demonstrate that nanoparticles are mostly spherical and oval in form, with diameters ranging from 10 to 50 nm, using the extract of *Malus domestica* fruit (Rosaceae) as a capping agent in another approach to manufacture sphere-shaped silver nanoparticles having a normal diameter of 20 nm [36,37]. UV-Vis spectroscopy is utilized to investigate nanoparticle production, whereas TEM and XRD are used to confirm different stages and shape, and FTIR is used to categorize organic components that role in nanoparticles stabilization and reduction. Silver nanoparticles are produced by reducing the aqueous solution of AgNO_3_ with latex of *Jatropha curcas* (Euphorbiaceae) [38]. The extract of *Sennaau riculata* leaves (*Cassiaauriculata*, Fabaceae) was also used as a capping agent in the production of silver nanoparticles. It was confirmed that the extracellular production of silver nanoparticles (AgNPs) using *Geranium* sp. leaf extract (Geraniaceae) that includes AgNO_3_ and has fast degradation of silver ions, results in the formation of stable silver nanoparticles with 40 nm diameter [39,40].

The leaf extraction of *Ficus benghalensis* (Moraceae)used for the synthesis of spherical stable silver nanoparticles (AgNPs) range from 10 to 50 nm in size. Different analytic techniques such as SEM, TGA, and XRD FTIR were used to measure the characteristics of silver nanoparticles [41]. The capping agent used in the preparation of silver nanoparticles is *Acorus calamus* extract (Acoraceae)that has antimicrobial and high antioxidant potential [42]. Other studies show that the TEM and XRD results indicated that the synthesized silver nanoparticles from *Boerhaavia diffusa* (Nyctaginaceae) extract show a spherical shape and a distinctive size of roughly 25 nm [43,44].

Various nanoparticles have been used against different bacteria, such as *Pseudomonas fluorescens*, *Aeromonas hydrophila*, *Flavobacterium* sp. *Acalypha lanceolata* leaf extract (= *A.indica*, Euphorbiaceae) is also used to synthesize silver nanoparticles (AgNPs) [45]. Moreover, silver nanoparticles were synthesized from *Ficus carica* leaf (Moraceae) and *Oleaeuropaea* (Oleaceae), and were characterized using XRD and SEM FTIR [46,47]. The *Abutilon indicum* extract (Malvaceae) was prepared for the synthesis of spherical silver nanoparticles and their antimicrobial potential against test pathogens such as *Streptococcus typhi*, *Staphylococcus* *aureus*, *Escherichia coli* and *B. subtilis* was determined [48].

Similarly, silver nanoparticles were also synthesized from *Aloe vera* (Xanthorrhoeaceae) that vary in size and shape [49]. Green spherical nanoparticles from *Sambucusnigra* (Adoxaceae) and *Syzygium* sp. fruit extract (Myrtaceae) were also synthesized [49], and XRD analysis revealed that they were crystalline. AgNPs were also synthesized using an extract of *Artocarpus heterophyllus* seed powder (Moraceae) [50]. Biogenic method is used for the manufacturing of silver-based NPs from *Boerhaayia diffusa* extract (Nyctaginaceae), which helps as a reducing and capping agent [51]. Silver nanoparticles as a colloidal solution exhibited maximum absorption at 418 nm in the UV-visible spectrum. A cubic structure with 25-nm particle size is shown by TEX and XRD analyses. Silver-based nanoparticles from *Leptadenia reticulate* leaves extract (Apocynaceae) arecrystalline and spherical, and are evaluated at 50–70 nm [52]. Mulberry leaf extract (*Morus* sp., Moraceae) was utilized to generate spherical and mono dispersed silver nanoparticles (AgNPs) having a particle size of 20 nm [47]. Numerous studies explain the production of silver nanomaterials by reducing the silver nitrate solution by means of olive leaf extract. Additionally, these nanomaterials showed potential against drug-resistant microbes [53]. During the investigation of nanomaterial characteristics using SEM, XRD, and TGA, it was confirmed by the results that nanomaterials are usually spherical, having a regular diameter ranging from 10 to 25 nm. In the greener method of manufacturing silver-based nanomaterials, researchers also use *Alternanthera ramosissima* (=*A. dentata*, Amaranthaceae) plant extract as a coating agent [43,44]. Silver-based nanomaterials exhibiting a size range of 38–72 nm were isolated from *Acacia leucophloea* (Fabaceae) [54].

## 3. Cost of Production of AgNPs

From 2021 to 2030, the silver nanoparticles market is expected to increase at a CAGR of 15.6 percent, from $1.5 billion in 2020 to $6.6 billion in 2030. Because of their excellent electrical conductivity, optical characteristics, and antimicrobial capabilities, silver nanoparticles (nanosilver) are one of the most extensively used nanomaterials. Particle composition, size distribution, surface chemistry, size, shape, coating/capping, particle morphology, dissolving rate, agglomeration, ion release efficiency, and particle reactivity in solution all influence silver nanoparticle biological activity (Global Opportunity Analysis and Industry Forecast 2021–2030). Furthermore, these initiatives are viewed as low-cost techniques that allow for the avoidance of toxic-producing items while also benefiting agricultural activity. One kilogram of silver nanoparticles (AgNPs) is projected to cost roughly USD 4 million, while one kilogram of raw silver costs around USD 14,000 [55,56].

## 4. AgNPs in Soils

Biological indicators are significant factors for assessing quality of the soil because the soil microbiota is involved in a variety of ecosystem activities, including inorganic matter breakdown and nutrient cycling. As a result, any change in soil microbial biomass has an influence on soil sustainability [57]. Needless to mention, the increased use of AgNPs due to their antibacterial action has resulted in their deposition in soil ecosystems, impacting the quality of the soil [58]. Several researchers have found that the hazardous effects on microbial communities are strongly reliant on their concentration in the soil, despite the fact that their environmental impact on the soil microbial community is still being studied [59]. According to Dinesh et al., NPs can affect soil in a variety of ways. To begin, their features, such as antibacterial activity, could have a direct influence on soil microbiota via creating reactive oxygen species (ROS). Second, NPs have the potential to alter the bioavailability of poisons and nutrients. Finally, indirect effects could arise from their interaction with naturally occurring organic hazardous chemicals, potentially increasing their toxicity [58]. Furthermore, various studies have demonstrated that AgNPs affect soil microorganisms that support plant growth and nutrient cycling, such as rhizobacteria, Pseudomonas fluorescence, Pseudomonas putida, and others. A major source of worry is the inhibition of denitrifying bacteria, with studies showing that at concentrations of 100 mg of AgNPs/kg of soil, there is a complete loss of these communities with no recovery signals, resulting in reduced nitrate to nitrogen conversion [60]. Meier et al. expressed concern that anthropogenic activities could affect soil ecosystems, causing microbial health to decline [61]. They tested the previous by exposing freshly harvested sandy loam soil to Ag nanoparticles solutions ranging from zero to 2000 mg/kg. After that, they added genomics-based assays to standard soil microbial analysis by measuring changes in community taxonomy structural and functional using 16S-rDNA profiling and metaproteomics. AgNPs influenced bacterial taxonomic structure as well as genes implicated in heavy metal resistance, according to the researchers. Furthermore, their presence caused a significant upregulation of several toxin response pathways [61].

## 5. AgNPs Role in Tolerance to Abiotic Stress

**Figure 4:**
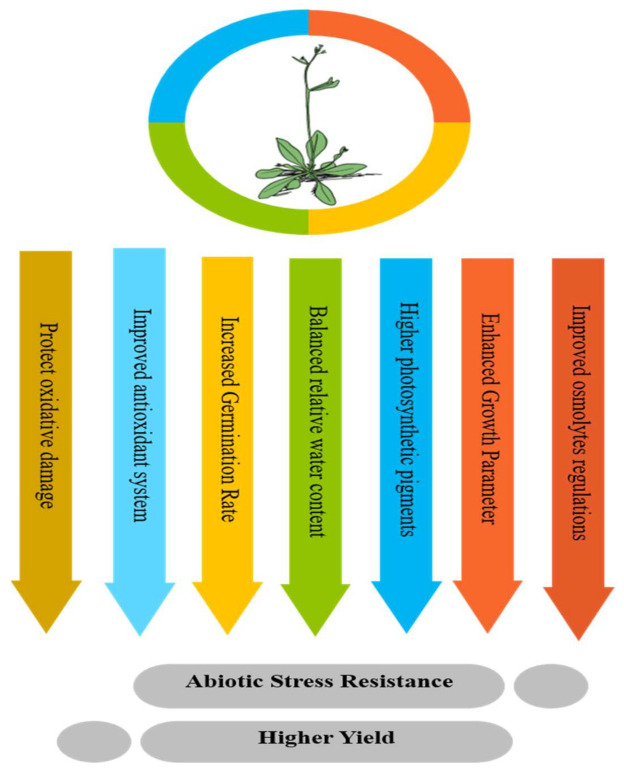
A schematic model figure shows the application of biogenic AgNPs tolerance to abiotic stresses (heat stress drought, salt, flooding, and chilling) in plants.

Various effects of abiotic stresses, that is, drought, salinity, and low/high temperature, have increased in the global ecological system day by day. In the last several years, nanotechnology has raised the focus of scientists in a diversity of research areas. Due to their nano-size, these nanomaterials had generated various distinct features that differentiate them from their macro-sized counterparts. Compared to larger substances, nanomaterials had a larger surface area, solubility, and reactivity. Hence, these materials are capable of achieving the objective of favorable role in recovering from adverse effects caused by these stresses and sustainable agriculture worldwide. The application of biogenic AgNPs in agriculture is gaining focus because of their stress-tolerant capability. Different forms of biogenic silver NPs have been tested for their probable role in the defense system against abiotic stress [62]. These nanomaterials enhance stress tolerance in crops by enriching nutrient absorption, improving enzyme reactions, and supporting the connection of plant roots with growth-promoting bacteria during abiotic stress (Figure 4). These primary investigations are promising and lead to a new period of nanomaterial application to enhance crop yield during harmful environmental circumstances.

## 6. Biogenic Silver Nanoparticles in Alleviating Drought Stress in Plants

Water is a fundamental element for plant survival as it support in transportation of nutrients, reduce drought stress, and make plant strong enough for better growth and development. Less availability of water is one of the most important abiotic stresses that show an adverse effect on plant growth and yield [16]. For better production, enough water is necessary in the soil for short to long space transportation, cell expansion through the plasma membrane, and osmoregulation [63]. Moreover, drought stress has a damaging impact on H_2_O movement in plants, however, it could be controlled through the opening of aquaporins (water permeable) [16]. To protect food safety, it remains precarious to minimize water shortage stresses and generate tolerance cultivars. However, numerous examinations discovered that biogenic AgNPs efficiently reduced salinity, but there was little literature emphasizing the combination of silver nanomaterials with drought [64,65]. A single investigation stated that silver-based nanomaterials helped maintain the water balance in lentils during conditions of water shortage stress by improving growth, length and weight of the plant. Additionally, the results of these investigations stated that the application of biogenic AgNPs improved germination during drought in lentils [66].

Nanotechnology is a rapidly evolving technology with the most recent updates and developments in a wide range of fields. However, nanotechnology applications and the use of nanoparticles in agricultural development and sustainable agriculture are still in their juvenile phase. As a result, to maximize the potential benefits of the unusual and unique qualities of nanoparticles in agriculture, it has become important to develop a fundamental understanding of the interaction of nanoparticles with plants at both the molecular and cellular levels [67].

## 7. Biogenic Silver Nanoparticles in Alleviation of Heat Stress in Plants

The increase in temperature of planet Earth, because of anthropogenic activities, is an important concern for mankind. High temperature or heat stress severely affects plant growth of compromising the quality of the ecological system and food security globally. Physiology, plant growth and the final quantity of eatable goods are influenced by heat stress to a limit that highlights physical injuries, physiological disorders, and biochemical changes acquired at certain stages of growth. To overwhelm the negative effects on crops of heat stress and other biotic and abiotic stresses, certain methodologies such as genetic engineering, hybridization, and QTL mapping are being used lately. In addition, these techniques had certain handicaps in the application, were expensive, and require technical expertise. In this situation, there is always a need for an affordable, feasible, and practical method that could suppress these handicaps [51,68,69,70,71]. In the agricultural sector, nano-biotechnology can be engaged for increased crop yield in adverse situations [72,73,74]. Among numerous monometallic NPs, biogenic AgNPs are frequently applied because of their characteristics such as magnetic, electrical conductivity, catalysis, optical polarization, and SER scattering [75]. Moreover, plant-derived silver nanomaterials play a role in increasing the dry weight and area of aerial parts. The effectiveness of these nanoparticles is examined by their size, reactivity, surface cover, chemical composition, and the concentration applied, making them more effective [76,77]. Silver nanoparticles prepared by the greener method also improved seed sprouting and growth of saplings of numerous plants such as *Boswellia ovalifoliolata* (Burseraceae), growth parameters such as plant length, and leaf area of *Zea mays* (Poaceae), *Phaseolus vulgaris* (Fabaceae), and *Brassica juncea* (Brassicaceae) [78,79].

The applications of biogenic silver nanoparticles in (*Triticum aestivum*) crops during high temperature were examined and physiological growth with respect to membrane stability index, chlorophyll contents, and relative water contents was analyzed. Heat stress considerably decreases these attributes such as MSI–16.3%, TTC–9.9%, and RWC–13.2% compared to the control. In plants, oxidative stress is one of the main causes of the disturbance of the cell membrane and biological molecules, leading to the reduction of plastid-chlorophyll pigments. An important increase in MSI–26.5%, TTC–19%, and RWC–12.2% was recorded in response to the application of AgNPs (50 mg/L) [26]. An increase in physiological growth is due to the alternation of cell meristematic activities (division and elongation). This growth is directly related to the protection of the cell membrane and enzymes or proteins, thus contributing to the tolerance heat [26]. Osmolyte generation adjusts less H_2_O potential by enhancing water absorption in a stressful environment [80]. The decrease in MDA level and electrolyte leakage in tobacco *(Nicotiana tabacum)* is due to the exogenous treatment of silver-nanomaterials [81]. A significant increase in carotene content and chlorophyll occurs in *Chlorella vulgaris* (Chlorellaceae) by the application of Ag-nanoparticles, which is due to a decrease in an alternative disturbance in chlorophyll pigment, resulting in better development [65]. The diversity in chlorophyll contents due to the application of these nanoparticles is also studied by various other plant scientists. Likewise, the contact of plant-based AgNPs remarkably increases photosynthetic pigments of *Oryza sativa* [82]. Silver-based nanoparticles significantly improve growth characteristics and photosynthetic potential along with the equilibrium of plant hormones. It is recommended that these nanomaterials may have a role in improving tolerance in plants during stress conditions [65].

Ag-nanoparticles prepared by a greener approach play a vital role in amending the adverse impact of high temperature when *Triticum aestivum* were treated by improving osmolytes level and generation of enzymes (antioxidant and non-antioxidants), as well as reducing the MDA level, LPX, and hydrogen peroxide concentration. Additionally, these nanoparticles have the efficiency to overcome oxidative stress in (*Triticum aestivum*), because of their distinctive optical and Plasmon resonance scattering features during high-temperature. A specific amount of biofabricated Ag-nanoparticles might have been applied to wheat, stimulating resistance in them against heat stress. The application of these nanoparticles in the vegetative and anthesis stages is vital to yield and growth during stress [6]. Hence, for the upcoming time, this eco-friendly, biologically prepared Ag-nanoparticles could be used in several acute high-temperature regions of the earth for substantial increase in tolerance from high temperature in wheat.

## 8. Biogenic Silver Nanoparticles in Alleviating Salinity Stress in Plants

Salt stress is an indispensable issue which is pertinent to note because an imbalance in ionic and osmotic balance affects homeostasis, oxidative system, and nutrient availability, ultimately reducing crop yield. In contrast, production regions are increasing in salt-affected regions due to extreme stress to meet food security targets to encounter the prerequisites of the constantly growing human population. The supply of nanoparticles to plants can considerably relieve the deleterious effects triggered by numerous harsh conditions, including salt stress, and therefore regulate adaptive mechanisms in plants [83]. Agricultural soils worldwide facing the salt stress are of great concern, and scientist are trying to evaluate the effectiveness of silver nanoparticles for growth under salt stress [84]. Salt stress damaged the yield of many crops, negatively affecting their growth at 125 million hectares. Through anthropogenic activities, 76 million in total are affected, and the remaining 1.5 million hectares of land are misplaced due to salinization and sodification per year [65]. Therefore, the harmful effect of such stresses on plant growth and development must be decreased by developing new technologies. Exposure of plants under salt stress to silver nanoparticles showed an improvement in their sodium chloride and potassium contents. Constant exposure to silver nanoparticles can be used to overcome damaged plants caused by varying salinity environment, and it was noticed that silver nanoparticles are much more stable in low salinity waters. Researchers are trying to ensure plant propagation in the field area; therefore, the productions of many transgenic plants are in progress. A method used to support plants in field conditions is the treatment of seeds with nanoparticles before sowing [85]. And silver nanoparticles also enhance wheat grain germination [65].

In addition to the importance of silver nanoparticles in improving the rate of seed germination, reactive oxygen species (ROS) are also produced in plant cell organelles such as chloroplast, plasma membrane, peroxisomes, and mitochondria under both natural and stress environments in tobacco plant [64]. Overproduction of ROS (reactive oxygen species) leads to harmful effects on genotypes, growth, development, and increases stress in plants. The combination of the treatment of plant AgNPs and NaCl decreased the production of TBARS (thiobarbituric acid reactive substances), H_2_O_2_ (hydrogen peroxide) [86]. An increase in the antioxidant potential of plants has been reported in response to adverse effects of salt stress [19]. Various enzymes are recognized to having their function in plants against oxidative stress such as anthocyanin, ascorbic peroxide, betaine, catalase, dehydroascorbate reductase, glutathione reductase, glycine, monodehydroascorbate reductase, proline, and superoxide dismutase [87]. Previously plant-based Ag nanomaterials have been shown to activate antioxidant defense systems in plants, resulting in decreased stress [86].

In soil, salinity is a worldwide problem and there is less knowledge in the literature on the effectiveness of Ag-nanomaterials in plants under salinity conditions [19]. Salinity leads to massive destruction due to an adverse effect on crop yield and growth. This condition is the main issue in the area of more than 1000 million-hectares, of which 76 million are pretentious by humankind, leading to more than 1 million hectares of agricultural land lost to salinization every year [65]. Ultimately, novel techniques are needed to reduce the harmful effects of these stresses. In plants, salt concentrations exposed to AgNPs significantly improved sodium, chloride, potassium, and osmolality. The consistency of silver nanoparticles can be managed by altering the salt level in aquatic surroundings, and it was noted that these NPs are more consistent in less salty water. More salt concentration could be harmful to plant growth and production [88]. Researchers have worked to promote plant germination under cultivation areas until growth management and the generations of novel transgenic varieties have become noticeable. Seed priming before plantation is one method for the promotion of plant germination on a large scale of cultivation. The priming with Ag-nanomaterials results in the germination and production of grains [89]. Moreover, the rate of seed germination increases with the application of silver NPs in tomatoes [90].

During normal and stressful situations, ROS generation in certain components of the cell occurs, such as cell membranes, chloroplasts, peroxisomes, and mitochondria. When ROS is overproduced in plants, it is interlinked with oxidative disruption and is affected by genetic makeup, level of development, and salinity participation. Compared to sodium chloride-treated samples, the linked silver nanoparticles and sodium chloride reduce H_2_O_2,_ TBARS, and electrolyte leakage [86]. Antioxidant defense increases in plants due to adverse impacts of salinity [19]. Numerous antioxidant enzymes play roles in defensive systems, such as ascorbate peroxidase, anthocyanin, catalase, dehydro-ascorbate reductase, glutathione reductase, glycine, and superoxide dismutase [87]. Previous research has shown that salinity could be minimized by silver nanomaterials by activating antioxidant mechanisms. Generally, the work explained and emphasized favorable techniques in Ag-nanomaterials that interacted with salinity tolerance, highlighting that the methods of persuading tolerance are depended on the antioxidant defense mechanism, ion accumulation, and proline metabolism [86].

The salinity environment affects the content of chlorophyll, sugar, lipids, and anthocyanin carotenoids [91]. Under high salt stress, plants have evolved multi factorial techniques to overcome such stresses with many anti-oxidative enzymes, among which CAT (catalase), SOD (superoxide dismutase), and POD (peroxidase), which are presumed to play an important role in ROS (reactive oxygen species) detoxification. CAT (catalase) and POD (peroxidase) are together involved in the conversion of H_2_O_2_ to O_2_ and H_2_O [92]. Function of proline, glycine betaine, polyols, and trehalose particles is analyzed. Under salty conditions, proline plays a role in overcoming osmotic stress in plants [64,93].

Various concentrations of silver nanoparticles in *Triticum aestivum* (Poaceae) increased fresh and dry weight, soluble sugar, total chlorophyll content, and antioxidant enzymes under salt stress [26]. Application of six impacts of silver nanoparticles on Plant 122 of 0–10 mg AgNPs in *Lathyrus sativus* (Fabaceae) improved seed sprouting, root and shoot length, and proline content under salt stress [66]. In *Thymus vulgaris* and *T. Daenensis* (Lamiaceae) germination percentage, seed vigor, and root and shoot length increased after exogenous application of 0–10 mg AgNPs under salinity stress [94]. Furthermore, different concentrations of AgNPs improved plant height, number of branches, and diameter and weight of fruits [95]. About 20 mg/kg AgNPs improved the percentage of germination, the length of the rootlets, and the fresh weight under salinity stress [96]. Low concentrations of silver nanoparticles improved the germination rate at various concentrations of NaCl (5, 10, 15, and 20 dS/m) [66].

## 9. Other Important Abiotic Stresses and Plant-Based AgNPs

Foliar applications of biogenic AgNPs have been reported to make plants tolerant toward cryostresses. The study revealed that a low concentration of AgNPs (0.25, 1.25 mg/dm^3^) showed a positive influence on green beans, resulting in a fast germination rate of plants in vitro and vivo and also showed improved height, dry and fresh weight, and photosynthesis of plants [97]. Photosynthesized AgNPs are reported to play a vital role in eliminating the deteriorations of high temp stress deterioration in wheat plants by reducing the contents of MDA and H_2_O_2_ and inducing an improved antioxidant defense system [6] (Figure 1).

## 10. Biogenic Silver Nanoparticle in Alleviation of Microbial Diseases in Plants

Nanobiotechnology is a developing new field of biotechnology having incredible potential beneficiaries against harmful microbes and food packaging (ecologically favorable). Metal-based nanomaterials have been comprehensively evaluated and often utilized in diversified fields due to their greater antimicrobial, biochemical, and physiological efficiencies [98]. The general antimicrobial potential of biogenic AgNPs is assumed to be influenced by its amount, relative size, surface area, and rate of release. The larger surface area and amount of Ag aid to enhance bacterial interaction by attaching to the protein (SH category). This resulted in the decline of bacterial activities and led to its inhibition. Additionally, the restriction of respiration and the transfer of electrons in the plasma membrane lead to death of the bacterial cell [99]. Silver has been used on a large scale as an antimicrobial agent as a food preservative and to store medical and therapeutics since very old times. It is also used to treat wounds and burns and also as an antiseptic [100,101]. The latest research has explored the facts that biogenic AgNPs are effective in the case of viruses, bacteria, and fungi.

**Figure 5:**
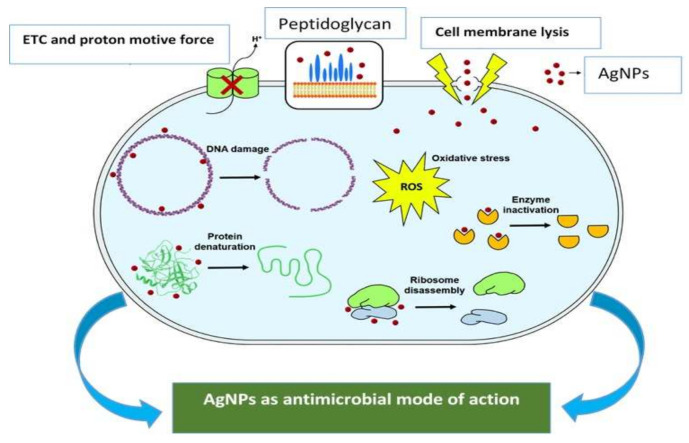
Plant-mediated AgNPs against various pathogens: representation of AgNPs as antimicrobial mode of action.

Gram-negative bacteria compared to Gram-positive bacteria are more effective because of their dense peptidoglycan layers in the cell wall that make the cell walls rigid, causing difficulties in NP penetration [102]. Phytosynthesized AgNPs using banana peel extract showed greater effects against *P. aeruginosa* and *E. coli* (Gram-negative bacteria) compared to *S. aureus* and *B. subtilis* (Gram-positive bacteria) [103]. However, these NPs were less effective against fungus *Candida* *albicans* (Figure 5). In the latest studies, phytosynthesized plant-based AgNPs were reported to show enhanced antimicrobial activities compared too chemically and physically synthesized NPs. It is also reported that phytosynthesized AgNPs showed greater antibacterial activities in the case of some human pathogens and lactic acid bacteria than synthetic antibacterial agents (Chloro hexidine) [104]. There are various factors (pH, test microorganisms, temperature, concentrations, shape and size) that define the antibacterial activity of AgNPs [105]. Various mechanisms of AgNP antimicrobial activity are illustrated in Figure 5. Their antimicrobial properties are related to the release of Ag+ ions, which bind to the negatively charged functional groups (carbonyl, amino and phosphate, sulphohydryl) found in membrane proteins, followed by alteration in structure and then increasing membrane permeability. These NPs induced a disturbance in the cell transport system resulting in cell death [44,106,107]. Furthermore, Ag enters the microbial cells and causes damage to their RNA, DNA, and peptide forming compounds and metabolic enzymes that inhibit cell division (translation, transcription) and respiration.

Another mechanism involved the accumulation of biogenic AgNPs in the membrane and the cell wall [108,109]. They induce changes in morphology following shrinking of the cytoplasm, detachment of the membrane, and resulting in rupturing of the membrane. As Ag+ ions do, AgNPs also show penetration in cells of bacteria and bind to biomolecules, following hanging of cell division and then cell death [108,110].

Biogenic AgNPs showed negative impacts on bacteria *Ralstonia solanacearum* by damaging cell envelopes, pit formation, and cell bulging. Loss of cell metabolic processes and surface adhering was also reported at 400 Lg AgNPs mL^−1^ [111]. NPs are also constructive compared to common antibiotics, as they have the ability to damage cells and do not allow microbes to develop immediate resistance [112,113]. Photosynthesized NPs are also ecofriendly and biocompatible and are also shielded by secondary metabolites that are basically part of the plant body and allow them to perform their function as an aspirant to be used as antimicrobial drugs [43]. AgNPs are the most extensively reported NPs, and in ancient times silver salt has been used as an antimicrobial agent. At present, phyto-synthesized AgNPs have captivated a profound compact of apprehension as antimicrobial activity, especially against the agents causing bacterial diseases [114]. The phytosynthesized AgNPs result in the destruction of the bacterial membrane and cause a barrier to enzyme function, leading to cell death [115]. AgNPs also modify the function of membrane proteins, resulting in leakage of cytoplasmic material leading to cell death [116].

Green synthesized AgNPs have the potential to generate ROS in case of oxidative stress in bacterial cells, which interprets the respiratory enzymes and leads to cell death [116]. *E. coli* and *S. aureus* are the pathogens that cause common infections, but AgNPs synthesized using *Berberis vulgaris* extract (Berberidaceae) have been reported to function against these infectious strains and their antimicrobial potential makes them an effective antimicrobial agent [117]. Another study reported the antimicrobial properties of AgNPs synthesized using the aqueous extract of *Mentha pulegium* (Lamiaceae) against several bacterial strains (*E. coli*, *S.pyogenes*, *S. aureus*, *C. albicans*) [118]. In another study, AgNPs synthesized from *Syzygium jambos* (Myrtaceae) showed antibacterial and antifungal properties [82]. AgNPs mediated by *Cucurbita maxima* (Cucurbitaceae), *Moringa oleifera* (Moringaceae), and *Acorus calamus* plant extract showed activity against broad spectrum microbes, for example *Bacillus subtilis*, *Pseudomonas aeruginosa*, *Escherichia coli*, and *Vibrio cholerae* [119]. With the tremendous antimicrobial potential of biosynthesized AgNPs, it is believed that plant extract-mediated AgNPs can be a tremendous nanoproduct to destroy these devastating plant pathogens. Therefore, there is a need to discover the application potential of green synthesized AgNPs besides distressing plant pathogens to defend the plants.

## 11. Biogenic Silver Nanoparticle in Alleviation of Cold Stress in Plants

The increasing occurrence of low temperature is also a major concern for farmers. Cryostress (0–15 °C) occurs at low temperature causing damage to cells without the formation of ice crystals in plant tissues, while the formation of ice crystals in cells leads to the death of crops called freezing stress which occurs at (<0 °C) [120]. Cryostress causes injury to the cell and leakage of electrolytes and also loss of fluidity; it also reduces germination, inhibits growth, and loss of crop yield [121,122]. Although its effects vary greatly between species and cultivars, tolerant species showed low membrane injury than sensitive species [123,124]. Various crops experienced extreme low temperatures in various areas of the world [125], and are exposed to limited water availability due to the uptake of distressed water at low temperatures [126,127]. This exposure of plants to chilling hinders plant growth and, therefore, is harmful to yield [127].

Chilling stress decreases the kinetics of numerous functional and metabolic processes that occur in plants [128,129]. It rigorously decreases the frequency and consistency of germination, seedling strength, and delays in genic plant development [130,131], which cause a loss in crop yield [128]. Recent research proved the role of silver nanoparticles in improving *Solanum lycopersicum* (= *Lycopersicon esculentum*, Solanaceae) seed germination under high salinity stress [64]. Thermal stress is classified into low temperature stress as freezing stress (<0 °C) and chilling stress (<20 °C) and high temperature stress according to environmental temperature. Low-temperature stress also lemmatized the topo-geographical distribution of plant species [132,133].

Various plant genes have mechanisms to deal with biotic and abiotic stresses. Due to variations in signal transduction pathways and metabolism processes, cryo-stress-related mechanisms are more complex [134]. The present day research on cold stress in plants is based on the model plant *Arabidopsis thaliana* (Brassicaceae), which has been extensively studied [135]. Furthermore, many studies have been reported in different plant species to discuss gene regulatory networks and molecular mechanisms [136]. Cryostress modifies the gene expression to about quarter of the total in the *Arabidopsis thaliana* genome and also showed an oppressive effect toward the expressions of genes and various metabolic pathways.

## 12. Silver Nanoparticle in Mitigation of Heavy Metal Stress in Plants

In recent years, heavy metal contamination is a global cause of concern and its pollution has hazardous effects on the environment and human health. It is known as the most dangerous threat to the sustainability of the agro-ecosystem [137,138,139,140]. The accumulation of heavy metals in the environment caused phytotoxicity; they are harmful to any kind of life, even if present in a small amount [141]. The accumulation of heavy metals in plants cells is a major cause of cessation of growth and even plant death as a result heavy metals are released into the environment by volatilization. Heavy metals also have an effect on plant physiological activities, morphological characteristics, the biochemical nature of plants, and reduce crop yield [142]. A total of 53 elements are classified as heavy metals among various naturally occurring elements and these include nickel (Ni), silver (Ag), zinc (Zn), lead (Pb), platinum (Pt), and arsenic (As), etc., and most of them do not have any essential function in plants. These are classified as essential elements (Cu, Zn, Fe, Mn, Mo, Ni, and Co) that play an important role in plants and their deficiency causes a delay in plant growth and development, while other are non-essential elements (Cd, Pd, Hg, Cr, As, and Ag) that lead to toxic effects on plants [143].

The continuous increase in population, globalization, and development has unconventional masses of wastewater that are gradually used as a water supply in urban agriculture regions. Watering with this leftover water takes a large amount of heavy metals and simply goes into the food chain by absorption of the soil and excessive plant uptake. The notable heavy metals are included in various significant physiological and biochemical processes of plants. Heavy metals play essential functions and are involved in the reaction of cell growths and additional molecular activities, as they are a fundamental part of many enzymes [144]. Generally, a plant germinates and grows normally when the amount of mineral nutrients provided contests the requirement of the plant. Deficiency of nutrients would result in signs resulting in mortality during severe conditions. The excessive availability of vital and non-vital heavy metals results in reduction and reticence of plant growth, affected by morphological and physio-biochemical changes [145]. More quantities of heavy metals also alter the storage, utilization, and transportation to other sites of essential metals available in plants. In addition, it harms biomolecules such as carbohydrate, fat, and protein matters of comestible plants, which germinate in soil containing heavy metals [146,147].

Heavy metal environmental pollution can excessively inhibit plant growth and increase the threat of harm to human and animal health through the biological amplification method [35,148,149,150]. In plants, the adverse influence of heavy metals is associated with retardation of physiological activities such as water retention, the use of minerals as nutrition and photosynthesis along with the excessive production of reactive oxygen species [151,152,153]. Heavy metal stress interjects metabolism, decreases growth by minimizing uptake of nutrients [68,154,155]. Furthermore, heavy metal stress damages plant mechanisms at the cellular level, such as decline in root growth, altering directing mechanisms and accumulation of reactive oxygen species, decline in the uptake of essential nutrients, and leads to membrane lipid peroxidation, and in extreme conditions leads to cell death of plants [156,157,158]. After the storage of heavy metals in grains and their introduction into the food chain, they can cause health problems in humans [159,160,161]. As soon as heavy metals were introduced into the environment, they are persevered there for countless years [162].

Heavy metals (Zn, Cu, Pb, Mn, Ni, Cr, Cd, As) which are transported through waste water and circulate in environment are serious threat to food. Some of them severely affect and damage plants and humans; however some have positive and important role in plant growth and developmental processes [151]. Remediation of metal-contaminated sites is rather challenging because heavy metals cannot be degraded by (photo) catalysis or chemical reactions [163]. Consequently, there are various methodologies for the recapitalization of heavy metals from waste water such as coagulation, membrane filtration, adsorption, electrolysis, and flocculation [164]. Adsorption is the most appropriate method, which is economic and has extraordinary efficacy in eliminating heavy metal ions from solutions. It has been extensively studied in the modern era and now there are different types of adsorbents that include activated carbon, root cell walls, and bamboo charcoal [165,166]. But they are not very effective and efficient, although there is a need to investigate more adsorbents with higher adsorption ability, economics, and faster kinetics [167]. Recently, nanoparticles with beneficial bacteria have been used in enhancing plants’ capacity to metal resistance. Strong research progress in the field of nanotechnology exposed the toxic and useful effects of nanoparticles on plant growth rate [168]. It was also revealed [169] that silver nanoparticles did not show harmful properties. Therefore, bioremediation, the use of nanoparticles in the elimination of toxic materials by activating the microbial action is used [142,170].

Biogenic nanoparticles have enormous applications, which is why tremendous attention is required by many researchers due to many specific properties such as large surface area, affluence of production, optical, magnetic, tunable surface chemistry and their functions that are different from bulk material [171]. It was also reported that rGO (reduced grapheme oxide) hybridized with magnetic and/or elemental silver nanoparticles (rGO/magnetite, rGO/magnetite/silver and rGO/silver) is a potential adsorbent for noxious heavy metals such as Cd, Ni, Zn, Pb, Cu, and Co. These nanohybrids (NHs) showed adsorption efficiency more than the sorbents already reported, for example, resins, activated biochar, hydrated nano-sized Zr oxide particles [172]. The effectiveness of nanoparticles was first experimented utilizing standard liquids of lead and cobalt, showing absorption wavelengths 406–458 nm and 406–445 nm for lead and cobalt metals, respectively, which explains why adsorption was carried out. This leads to higher removal efficiency for lead metal than for cobalt. Moreover, the effectiveness of NPs for the removal of metal ions from groundwater was studied utilizing plasma optical emission spectroscopy. The observations noted highlights removal effectiveness of 77% and 24% for lead and cobalt, respectively. It is significant to note that the quantity of metal ions decreases with increasing incubation timeframe [171].

The measurements of Hg in sediment poured water, combined through EXD analysis and SEM analysis revealed that addition of silver nanoparticles in pore water is stabilized and produced HgO which affect the movement of Hg to long distance [173]. This investigation was done to evaluate the effects of silver nanoparticles (AgNPs) on soils mobility and capacity of plant to uptake toxic heavy metals from wastewater used in irrigation. *Raphanus sativus* (Brassicaceae) was grown in controlled environmental conditions, irrigated with wastewater containing lethal metal with and without nanoparticles. Several previous studies claim that myriad of heavy metals (chromium, copper, iron, lead, and zinc) uptake is regulated by silver nanoparticles present in waste water and it is a great risk for human [174].

Remediation of heavy metals through photosynthesized silver nanoparticles. Atomic absorption spectroscopy was performed to test effluent samples from an industrial area, and out of four metals two (cadmium and chromium) are remediated by AgNPs through nano-fibrillated cellulose via the simulation method using metal salt concentration of 100 mg/L for 160 min. Biogenic AgNPs were observed to show the highest efficacy for cadmium, which was 83% and followed by chromium 47%. Due to the non-hazardous nature and biocompatibility of AgNPs, it is supposed that they can be used as an alternate method for the elimination of toxic heavy metals from the environment [175].

## 13. Antioxidant Potential of Silver Nanoparticles in Plants

Antioxidant activity has the ability to reduce the proportion of autoxidation process by reducing initiation events or interacting with chain-carrying radicals [176]. Different mechanisms are responsible for the antioxidant potential of nanomaterials, but in the case of biogenic silver nanoparticles (AgNPs) or other metallic nanoparticles, such as catalase mimic (CAT-mimic) is the major mechanism [176]. According to previous references biogenic AgNPs have the ability to decompose hydrogen oxide to the water molecule and oxygen (H_2_O_2_ to H_2_O and O_2_) on their surface, and have antioxidant activity [177,178]. Therefore, this is the main reason why most of the plant-based bio-synthesized silver nanoparticles (AgNPs) exhibit antioxidant activities. The activity of antioxidants represents a system of antagonism to oxidants. Antioxidant substances are naturally present and can also be synthesized in the laboratory to prevent and break the cell damage. Several NOS (nitrogen oxide species), RNS (reactive nitrogen species), and other unstable molecules cause cell damage. Silver nanoparticles (SNPs) have the ability to be used in food packaging, processing, and preservation [179].

The phytogenic silver nanoparticles are well-known for their outstanding antioxidant activity as shown in the previous literature, whereas the plant-based silver nanoparticles were well discovered to be able to rummage the 2,2-diphenyl-1-picrylhydrazyl (DPPH) and 2,2′-azino-bis(3-ethylbenzothiazoline-6-sulfonic acid (ABTS) free radicals in a short time period [180,181]. Numerous researchers presented a fascinating study of silver nanoparticles mediated by sweet potato that demonstrated antioxidant activity.

Furthermore, in a study it was also revealed that silver nanoparticles of *Taraxacum officinale* leaf extract (Asteraceae) had great antioxidant potential to reduce the damaging effects of reactive oxygen species (ROS). Free radicals are highly reactive entities created by cells during different activities such as respiration and immunological function, but their overproduction has negative consequences due to their interaction with biomolecules [179,182]. Plant-based SNPs’ high nitric oxide and DPPH scavenging action of plant-based SNPs make them a good source of natural antioxidant mediators [179,183]. These types of relationship are extremely harmful, causing atherosclerosis, aging, cancer, inflammation, and cardiovascular disorders. However, the silver nanoparticles synthesized by plants are highly rich in DPPH scavenging activity, nitric oxide, which allows them to be used as natural antioxidant mediators [179,183]. The high antioxidant potential of plant-based silver nanoparticles was investigated due to the diversity in their ABTS, DPPH, NO radicals and the interaction among phytochemicals extracted from plants through stabilization and green synthesis. Researchers highly recommended that plant-synthesized nanoparticles can play a vital role in the formation of new drugs as major antioxidant chemicals in pharmaceutical industries [184,185]. Plant-based silver nanoparticles are believed to be biocompatible while using them as alternative antioxidants in food storage and the preservation of medicines [186]. However, given the significance of green synthetic silver nanoparticles, it is not arbitrary to rely on the fact that the SNPs might be a viable option for different cosmetic sectors, such as anti-aging lotions and sunscreen agents. Plant-mediated silver nanoparticles have the potential to be worked in the treatment of the most severe diseases, including as osteoporosis, cancer, and coronary heart disease, which are triggered by oxidative stress that is generated by reactive oxygen species [15,187].

## 14. Conclusions and Future Prospective

**Figure 6:**
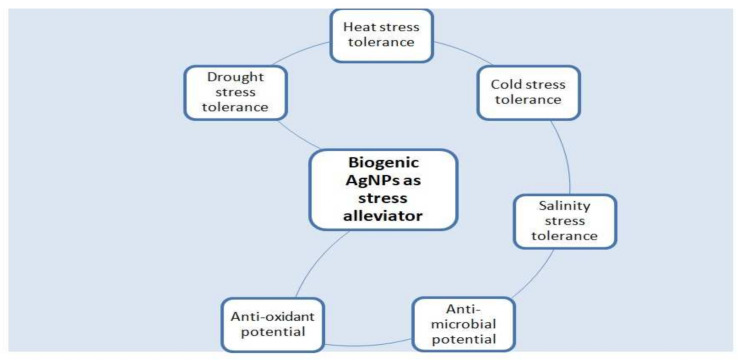
Plant-mediated AgNPs as a stress alleviator against agricultural plants.

Climate change, loss of water and land resources, energy issues, biotic and abiotic stressors, and other factors all pose significant threat to the global agriculture. To address these problems, appropriate solutions must be more sustainable and environmentally friendly. In this context, agri-nanotechnology is regarded as one of the most significant and promising problems (Figure 6). It is evident that biogenic AgNPs have beneficial and negative impacts on plants. Under abiotic stress, AgNPs can also enhance growth, chlorophyll content, photosynthetic efficiency, and ant oxidative defense mechanisms. In spite of being a much debated topic, the effects of plant-based silver nanoparticles AgNPs on plants have yet to be systematically examined. Major source of concern in environment now days are abiotic and biotic stresses. Major abiotic stresses i.e., drought, salt, and very high and low temperatures that influence plant growth and development, and biotic stresses such as microbial diseases also have negative effect on the plant growth.

A well-organized and established research program that evidently describes the appropriate nanoparticle dosages to employ for different crops under varied environmental circumstances is required. Without a question, there is still much to learn. Although this is a much debated subject, efficiency and effects on the plant derived silver nanoparticles must be examined systematically. Abiotic and biotic stresses are the main environmental concerns. Abiotic stresses such as drought, salt, low and high temperature, and water are affecting plant growth and development while biotic stresses such as microbial diseases also influence on plant growth and yield. The fundamental subject is the determination of new exploratory and investigative biomarkers to find harmful effects of silver nanoparticles on plants and their reduction. The geographical patterns of plant explored to green synthesized nanoparticles in response to abiotic and biotic stresses will be the main focus of future study. A detailed study is also required to examine the factors that influence interactions among plant genes in response to green synthesized nanoparticles. Moreover, hormone signals in response to abiotic and biotic stresses by plants should be focused in future studies along with the ultimate effects of silver nanoparticles. The hormonal transfer inside the cell parts and between the cells while signal transduction pathways in response to silver nanoparticles needs to be addressed in future. We hope that a full molecular and signaling exploration that addresses these and other necessary complications will provide a clearer picture of biotic and abiotic stress-resistant agriculture.

## Data Availability

All the obtained data are presented in this article.
